# Binding of *Campylobacter jejuni* FliW Adjacent to the CsrA RNA-Binding Pockets Modulates CsrA Regulatory Activity

**DOI:** 10.3389/fmicb.2020.531596

**Published:** 2021-01-11

**Authors:** Marek Bogacz, Faiha M. El Abbar, Claudia A. Cox, Jiaqi Li, Jarred S. Fiedler, Lynn K. H. Tran, Paul M. H. Tran, C. Luke Daugherty, Kate H. Blake, Zhirui Wang, Parastoo Azadi, Stuart A. Thompson

**Affiliations:** ^1^Division of Infectious Diseases, Department of Medicine, Medical College of Georgia, Augusta University, Augusta, GA, United States; ^2^Complex Carbohydrate Research Center, The University of Georgia, Athens, GA, United States

**Keywords:** motility, flagella, biofilm, regulation, flagellin

## Abstract

*Campylobacter jejuni* CsrA is an mRNA-binding, post-transcriptional regulator that controls many metabolic- and virulence-related characteristics of this important pathogen. In contrast to *E. coli* CsrA, whose activity is modulated by binding to small non-coding RNAs (sRNAs), *C. jejuni* CsrA activity is controlled by binding to the CsrA antagonist FliW. In this study, we identified the FliW binding site on CsrA. Deletion of the C-terminus of *C. jejuni* CsrA, which is extended relative to sRNA-binding CsrA proteins, abrogated FliW binding. Bacterial two-hybrid experiments were used to assess the interaction of FliW with wild-type CsrA and mutants thereof, in which every amino acid was individually mutated. Two CsrA mutations (V51A and N55A) resulted in a significant decrease in FliW binding. The V51A and N55A mutants also showed a decrease in CsrA-FliW complex formation, as assessed by size-exclusion chromatography and surface plasmon resonance. These residues were highly conserved in bacterial species containing CsrA orthologs whose activities are predicted to be regulated by FliW. The location of FliW binding was immediately adjacent to the two RNA-binding sites of the CsrA homodimer, suggesting the model that FliW binding to CsrA modulates its ability to bind to its mRNA targets either by steric hindrance, electrostatic repulsion, or by altering the overall structure of the RNA-binding sites.

## Introduction

*Campylobacter jejuni* is an important human pathogen, and one of the leading bacterial causes of acute gastroenteritis throughout both developed and developing areas of the world (WHO, 2015). *C. jejuni* infection is responsible for the deaths of 20,000–40,000 children each year, primarily in developing countries (GBD Diarrhoeal Diseases Collaborators, 2017). Additionally, ∼0.1% of cases of *C. jejuni* infection precede the development of Guillain-Barré syndrome, an acute paralytic autoimmune disease resulting from molecular mimicry of *C. jejuni* surface antigens to human gangliosides (Nachamkin et al., 2002). Thus, the global burden of disease due to this organism is enormous. Complicating therapy against *Campylobacter* is its rapidly increasing resistance to antibiotics, leading the US Centers for Disease Control and Prevention to classify *Campylobacter* as a serious antibiotic resistance threat (CDC, 2019).

*C. jejuni* genomes encode a relatively small number of regulatory elements compared to other enteric pathogens (Parkhill et al., 2000), but have the RNA-binding, post-transcriptional regulator CsrA. CsrA proteins bind AnGGA-containing stem loops in target mRNAs, particularly in their 5′ untranslated regions (UTRs), affecting their translation and/or mRNA stability (Babitzke and Romeo, 2007). In both *C. jejuni* and *E. coli*, the number of proteins regulated by CsrA can be quite large, with numerous effects on cellular metabolism and pathogenesis (Fields and Thompson, 2008; Edwards et al., 2011; Fields et al., 2016; Li et al., 2018). We previously demonstrated that *C. jejuni* CsrA regulates multiple processes associated with bacterial virulence, as a *csrA* mutant had impaired motility and ability to form biofilm, decreased resistance to oxidative stress, reduced epithelial cell binding and mouse colonization, as well as increased host cell invasion (Fields and Thompson, 2008). Some of these phenotypes could be a result of dysregulated translation of flagellin (*flaA*) mRNA, a known target of *C. jejuni* CsrA (Dugar et al., 2016; Fields et al., 2016; Radomska et al., 2016).

In *E. coli*, CsrA is a homodimer with the two subunits arranged in an antiparallel manner, with each subunit containing five beta strands (β_1_–β_5_). Interaction of β_1_ of one subunit with β_5_ of the opposing subunit forms two identical RNA-binding surfaces (Mercante et al., 2006, 2009). Site-directed mutagenesis (SDM) of both *E. coli* CsrA and *C. jejuni* CsrA elucidated a number of amino acids that affected RNA binding, with amino acid substitutions in β_1_ (amino acids 2–7) and β_5_ (amino acids 40–47) showing the most profound RNA-binding defects (Mercante et al., 2006; El Abbar et al., 2019). The activities of *E. coli* and closely related CsrA proteins are modulated by competitive binding to inducible small, non-coding RNAs (sRNAs) (Vakulskas et al., 2015). However, *B. subtilis*, *C. jejuni*, and some other CsrA-containing bacteria lack these sRNAs, and CsrA activity is instead regulated by protein-protein interactions with FliW (Mukherjee et al., 2011; Dugar et al., 2016; Radomska et al., 2016; Li et al., 2018). Mutation of *C. jejuni fliW* leads to altered expression of flagellar and non-flagellar proteins of the CsrA regulon, further demonstrating the regulatory effects of FliW on CsrA activity (Li et al., 2018).

The purpose of this study was to understand the mechanism by which interaction with FliW leads to altered CsrA activity, by identifying the site of CsrA to which FliW binds. Using a combination of deletion analysis, SDM, bacterial two-hybrid system, size-exclusion chromatography, and surface plasmon resonance, we determined that FliW binds adjacent to the predicted RNA-binding pockets of CsrA. Binding of FliW at this location likely prevents binding of CsrA to target mRNAs by steric hindrance, possible electrostatic repulsion, or by altering the structure of the RNA-binding sites of *C. jejuni* CsrA.

## Materials and Methods

### Bacterial Strains and Growth Conditions

The bacterial strains that were used in this study are listed in Table 1. *E. coli* strains were routinely grown in Luria-Bertani (LB) broth or agar at 37°C in an ambient atmosphere. Media were supplemented with antibiotics at the following concentrations when appropriate: ampicillin (Amp), 50 μg/ml; chloramphenicol (Cm), 30 μg/ml. *C. jejuni* was grown in Mueller-Hinton (MH) broth or agar at 42°C in a tri-gas incubator with an atmosphere of 10% O_2_, 10% CO_2_, and 80% N_2_.

**TABLE 1:** Bacterial strains and plasmids used in this study.

Strain or plasmid	Description	Resistance	Source or reference
**Strain**			
***Campylobacter jejuni***			
81–176	Wild type		Black et al., 1988
***Escherichia coli***			
DH5α	Cloning strain		Thermo Fisher Scientific
One Shot Top 10	Cloning strain		Thermo Fisher Scientific
BL21(DE3)pLysS	Protein expression strain	Cm	Promega
SP850	Bacterial two-hybrid host strain		Karimova et al., 1998
**Plasmids**			
pCRII-TOPO	Cloning vector	Amp, Km	Invitrogen
pT18	Bacterial two-hybrid plasmid expressing *cya*T18	Amp	Karimova et al., 1998
pT25	Bacterial two-hybrid plasmid expressing *cya*T25	Cm	Karimova et al., 1998
pT18-*csrA*	pT18 expressing CsrA-T18 fusion protein	Amp	This study
pT25-*fliW*	pT25 expressing T25-FliW fusion protein	Cm	This study
pET-20b-CsrA-His_6_	*csrA* cloned into pET-20b(+)	Amp	Fields et al., 2016
pET-20b-FliW-His_6_	*fliW* cloned into pET-20b(+)	Amp	Li et al., 2018

### Construction and Expression of CsrA C-Terminal Deletion Mutants

Sequential deletions of the C-terminal region of CsrA were constructed by using inverse PCR with the primers listed in Supplementary Table S1. PCR primers csrA-5.R, csrA-10.R, csrA-15.R, csrA-20.R, and csrA-25.R were used in conjunction with primer csrA-del.F on pET-20b-CsrA-His_6_ template (Fields et al., 2016), to generate derivatives lacking 5, 10, 15, 20, and 25 C-terminal amino acids of CsrA (designated CsrAΔ5, Δ10, Δ15, Δ20, and Δ25, respectively). In each case a stop codon was introduced to avoid translation of the His_6_ tag. Deletion mutants of CsrA were overexpressed in *E. coli* strain BL21(DE3)pLysS. Cells were grown at 37°C in LB broth containing 50 μg/ml Amp and 30 μg/ml Cm. When cells reached mid-log phase (OD_600_ = 0.5), the expression of the proteins was induced with 0.5 mM IPTG for 3 h.

### Protein Production and Purification

CsrA-WT-His_6_, CsrA-V51A-His_6_, and CsrA-N55A-His_6_ were purified as described previously (El Abbar et al., 2019). FliW-His_6_ protein was produced in *E. coli* strain BL21(DE3)pLysS. Cells transformed with pET-20b-FliW-His_6_ (Li et al., 2018) were grown in Luria-Bertani broth (LB) containing ampicillin and chloramphenicol at 37°C. When the culture reached OD_600_ = 0.6, the expression of the protein was induced with 0.5 mM IPTG for 4 h. Cells were collected by centrifugation and the cell pellet was resuspended in buffer A: 50 mM Tris-HCl, pH 7.4, 300 mM NaCl, 5 mM β-mercaptoethanol, lysed by passing twice through a French Press (Thermo), and centrifuged at 10,000 × g for 10 min. The pellet was subsequently dissolved in buffer B: 50 mM Tris-HCl, pH 7.4, 8 M urea, 5 mM β-mercaptoethanol, and the sample was centrifuged at 15,000 × g to remove insoluble material. Supernatant was mixed with Ni-NTA chromatography resin (Ni-NTA Agarose, Qiagen). After protein binding for 1 h in 4°C, the resin was washed 3 times with 10 resin volumes of buffer B and protein was eluted with buffer C: 50 mM Tris-HCl, pH 7.4, 8 M urea, 5 mM β-mercaptoethanol, 300 mM imidazole. The protein was then diluted to 1 mg/ml with buffer C and refolded by dialysis against buffer E: 50 mM Tris-HCl, pH 7.4, 300 mM NaCl, 0.4 M arginine, 1 mM EDTA, 1 mM glutathione, 0.1 mM glutathione disulfide, and subsequently against buffer F: 20 mM Tris-HCl, pH 7.4, 300 mM NaCl, 5 mM β-mercaptoethanol. Final protein purity was achieved by gel filtration on HiPrep Sephacryl S-100 HR column (GE Healthcare) in buffer F.

### Pulldown Assays With FliW and CsrA Wild Type or Mutants

Purified FliW-His_6_ protein in 50 mM sodium phosphate, pH 8.0, 300 mM NaCl, 0.01% Tween-20 was incubated with 10 μl of Dynabeads^TM^ His-Tag Isolation & Pulldown (Invitrogen) for 10 min, and the beads were subsequently washed four times with the same buffer. *E. coli* BL21(DE3)pLysS cells expressing wild type CsrA or CsrA proteins containing C-terminal deletions were resuspended in 20 mM sodium phosphate, pH 7.5, 150 mM NaCl, 0.01% Tween-20, and lysed by sonication. Insoluble material was removed by centrifugation at 12,000 × g and lysates were then incubated with FliW-coated beads for 20 min at room temperature. Beads were subsequently washed four times with 300 μl of the same buffer. Protein complexes were eluted from magnetic beads with 50 mM sodium phosphate, pH 7.5, 150 mM NaCl, 300 mM imidazole.

For Western blot analysis, samples were separated on a 15% SDS-PAGE gel and then electroblotted onto PVDF membranes (Millipore). Membranes were probed overnight with 1:200 dilution of primary antibodies against CsrA in TBS-T (Tris-buffered saline, 0.05% Tween 20). After washing twice with TBS-T, horseradish peroxidase-conjugated secondary goat anti-rabbit antibodies (Bio-Rad, 1:20,000 in TBS-T) were applied. Blots were washed twice and incubated in SuperSignal West Pico Chemiluminescent substrate (Thermo Fisher Scientific). Specific bands were visualized using G:Box imager (Syngene).

### Site-Directed Mutagenesis (SDM) and Bacterial Two-Hybrid System

To identify the FliW binding site of CsrA, we used a combination of SDM and the bacterial two-hybrid system described by Karimova et al. (1998) based on reconstitution of adenylate cyclase activity. We first performed SDM to create substitution mutations in which each of the 75 amino acids of CsrA was converted to alanine, with the exception of two native alanine residues (A30 and A36) that were converted to valines. A Q5 SDM kit (NEB) was used for all SDM experiments. For CsrA mutants at amino acid positions 40–60, *csrA* was amplified using PCR with the primers csrA-*Kpn*I F and csrA-*Hin*dIII R (Supplementary Table S1), using template DNA of CsrA point mutations previously cloned into pET20b (El Abbar et al., 2019). The PCR products were digested with *Kpn*I, *Hin*dIII, and *Dpn*I and cloned into pT18. To create mutations at CsrA amino acid positions 11–39 and 61–67, we used the appropriate SDM primers (Supplementary Table S1) with template DNA of c*srA* WT cloned into pT18. For CsrA mutations at amino acid positions 1–10 and 68–75, mutations were designed in the forward PCR primers for 1–10 and in the reverse PCR primers for 68–75. These primers were combined with PCR primers csrA-*Hin*dIII.R or csrA-*Kpn*I.F, respectively (Supplementary Table S1), and used to amplify *csrA* from *C. jejuni* 81–176 chromosomal DNA. Each PCR product was then cloned into pT18. The *fliW* gene was amplified from 81–176 chromosomal DNA using primers fliW-*Pst*I.F and fliW-*Bam*HI.R (Supplementary Table S1), and was then cloned into pT25 using *Pst*I and *Bam*HI. We performed bacterial two-hybrid experiments as described (Karimova et al., 1998) to detect the interaction of FliW with WT or mutant CsrA proteins. Briefly, the interaction of FliW with WT or mutant CsrA proteins was assessed by co-expressing CsrA constructs cloned into pT18 with FliW cloned into pT25 in the *E. coli* host strain Sp850 (Karimova et al., 1998). Co-expression of vectors pT18 and pT25 lacking inserts was used as a negative control. β-galactosidase assay (Miller, 1972; Karimova et al., 1998) was performed to measure the degree of interaction between CsrA and FliW.

### Analytical Size-Exclusion Chromatography (SEC)

Purified FliW-His_6_ protein was mixed with CsrA-His_6_ wild-type or mutant proteins in SEC buffer (20 mM sodium phosphate, pH 7.5, 150 mM NaCl, 5 mM MgCl_2_, 2 mM DTT) at an equimolar ratio for a final protein concentration of 1 mg/ml, and kept on ice for 30 min. 100 μl of each sample was loaded on a Superdex 200 10/300 GL column (GE Healthcare Life Sciences), and separation was performed in SEC buffer at a flow rate of 0.4 ml/min.

### Surface Plasmon Resonance (SPR)

#### Immobilization

For SPR analysis, FliW-His_6_ was immobilized to a Series S sensor chip CM5 (GE Healthcare Life Sciences) via primary amine groups. The immobilization was performed with a BIAcore T100 system (GE Healthcare Life Sciences) programmable method. Separate vials containing 200 μl of 0.1 M N-hydroxysuccinimide (NHS), 200 μl of 0.5 M 1-ethyl-3-(3-dimethylaminopropyl)carbodiimide hydrochloride (EDC), 70 μl of 1 M ethanolamine-HCl and an empty vial for mixing EDC and NHS were placed in the autosampler together with 100 μl of FliW in 10 mM sodium acetate buffer (pH 4.5). PBS P20 buffer, pH 7.4 was used as the running buffer. The immobilization cycle was performed at a flow rate of 5 μl/min.

#### Kinetic Assay

The assay was performed on a BIAcore T100 system (GE Life Sciences). 20 mM phosphate buffer containing 0.05% Tween 20, pH 7.4 was used as running buffer and sample buffer. The kinetic analysis used two flow cells to collect data over a concentration range of 0–500 nM. The association was made by injecting CsrA samples at a constant flow rate of 30 μL/min for 180 s, followed by 600 s of dissociation. The sensor chip surface was regenerated to remove bound CsrA by injection of 25 μL of glycine-HCl, pH 2.5 at a constant flow rate of 50 μL/min. All samples were filtered through a 0.2 μm filter. Sensograms were analyzed using a bivalent analyte binding model for interaction of a monovalent ligand with analyte molecules that carry two identical and independent binding sites.

### Sequence Alignment

CsrA (or its ortholog RsmA) proteins from diverse bacterial species were aligned using ClustalOmega at EMBL-EBI (Sievers et al., 2011; Li et al., 2015). *C. jejuni* (WP_002852854.1), *H. pylori* (WP_120911520.1), *W. succinogenes* (WP_011138784.1), *N. profundicola* (WP_127679601.1), *S. denitrificans* (WP_011372074.1), *T. pallidum* (WP_010882102.1), *B. subtilis* (WP_014478124.1), *C. difficile* (WP_022619991.1), *C. botulinum* (WP_003367280.1), *Geobacillus thermodenitrificans* (WP_011 888204.1).

### Modeling of CsrA-FliW Interactions

To model interaction of *C. jejuni* CsrA with FliW, we used the only available crystal structure of CsrA containing a C-terminal extension from *G. thermodenitrificans* [PDB: 5DMB (Altegoer et al., 2016)]. Molecular graphics and analyses were performed with UCSF Chimera (Pettersen et al., 2004).

## Results

### FliW Binds Within the C-Terminal Region of CsrA

**FIGURE 1:**
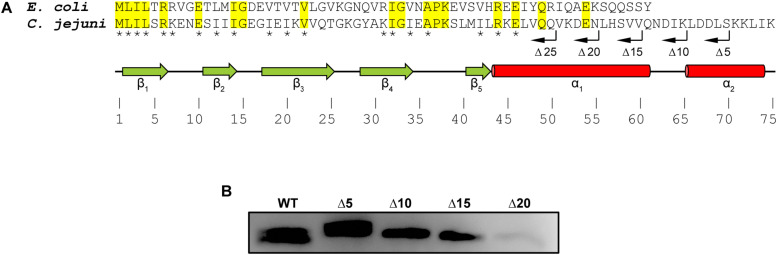
Mapping the FliW-binding region of *C. jejuni* CsrA. **(A)** CsrA proteins from *C. jejuni* and *E. coli* were aligned using ClustalOmega at EMBL-EBI (Sievers et al., 2011; Li et al., 2015). Identical residues are shaded in yellow. Asterisks indicate amino acids that are involved in RNA binding by *C. jejuni* CsrA (El Abbar et al., 2019). Green arrows and red cylinders indicate predicted β-strands and α-helices, respectively. β_1_ and β_5_ are involved in forming intermolecular RNA-binding interfaces within CsrA homodimer (Mercante et al., 2006). CsrA proteins containing sequential deletions of 5, 10, 15, 20, and 25 amino acids from the CsrA C-terminus (Δ5, Δ10, Δ15, Δ20, and Δ25, respectively) are indicated by bent arrows. **(B)** *C. jejuni* CsrA WT and mutant proteins shown in **(A)** were used in pulldown assays with FliW immobilized to magnetic beads. Bound CsrA proteins were detected with antibodies against *C. jejuni* CsrA. Wild-type CsrA and deletions of 5, 10, and 15 C-terminal amino acids retained FliW-binding ability, however, deletion of 20 amino acids from the C-terminus largely abrogated binding to FliW.

Alignment of CsrA proteins from *C. jejuni* and *E. coli* shows both conserved and divergent features (Figure 1A; Fields and Thompson, 2012). Both CsrA proteins are composed of five β-strands. CsrA from *C. jejuni* is longer (75 amino acids), and its activity is modulated by FliW (Dugar et al., 2016; Radomska et al., 2016; Li et al., 2018). In contrast, *E. coli* CsrA is shorter (61 amino acids) (Figure 1A) and its activity is regulated by binding to sRNAs (Jin et al., 2002; Thomson et al., 2006; Babitzke and Romeo, 2007; Fakhry et al., 2017; Janssen et al., 2018; Potts et al., 2019). As the C-terminus of *C. jejuni* CsrA is longer than the analogous region of CsrA proteins of *E. coli* and other bacteria, whose activities are regulated by sRNAs rather than by FliW (Fields and Thompson, 2012), and adjacent to the most C-terminal β-strand (β_5_) that is involved in RNA binding in both *E. coli* and *C. jejuni* (Figure 1A; Mercante et al., 2006; El Abbar et al., 2019), we hypothesized that FliW would bind to this region. We therefore used inverse PCR to construct a series of deletions in the *C. jejuni* 81–176 *csrA* gene cloned into an expression vector (Fields et al., 2016), resulting in a deletion of 5, 10, 15, 20, and 25 amino acids of the C-terminus (lacking a C-terminal His_6_ tag, Figure 1A). WT and C-terminally deleted proteins were expressed in *E. coli*, and cell lysates containing these proteins were used in pulldown assays with FliW-His_6_ immobilized on magnetic beads (Figure 1B). Bound CsrA was detected using anti-CsrA antibodies. CsrA-WT bound to FliW (Li et al., 2018), as did mutant CsrA proteins lacking the C-terminal 5, 10, and 15 amino acids (Figures 1A,B). However, the deletion of 20 amino acids from the C-terminus of CsrA almost completely abrogated binding, suggesting that at least part of the FliW binding site was located in the region around amino acid 56 of *C. jejuni* CsrA. A CsrA protein containing a 25 amino acid deletion was highly unstable and could not be used in these assays (data not shown).

### *C. jejuni* FliW Binds Adjacent to the Predicted RNA-Binding Pockets of CsrA

**FIGURE 2:**
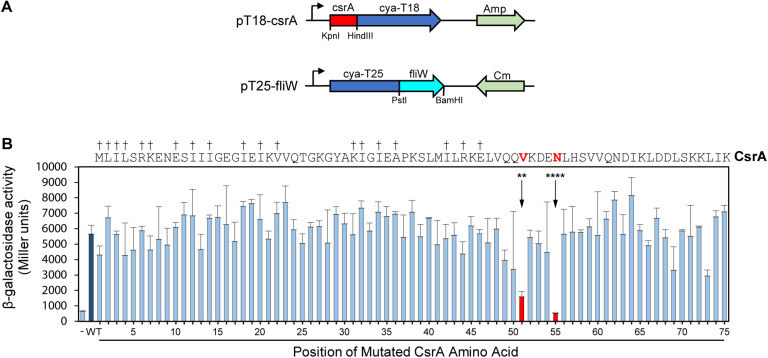
Bacterial two-hybrid assays define CsrA amino acids that play a role in binding FliW. A bacterial two-hybrid assay was used to demonstrate interaction between CsrA and FliW. **(A)** Translational fusions of CsrA and FliW to the T18 and T25 fragments of *B. pertussis* Cya were prepared for use in bacterial two-hybrid experiments. pT18-*csrA* (top) expresses Cya-T18 fused to the C-terminus of CsrA, and pT25-*fliW* (bottom) expresses Cya-T25 fused to the N-terminus of FliW. Amp and Cm were used for selection of the respective plasmids. **(B)** Site-directed mutagenesis was used to individually mutate each CsrA amino acid to alanine (two native alanine residues were mutated to valine). β-galactosidase activity indicates the degree of interaction. Negative control (co-expression of vectors pT18 and pT25 lacking inserts) is represented by first bar (-). Interaction between FliW and CsrA-WT is represented by dark blue bar (WT). CsrA mutants V51A (**p < 0.01) and N55A (****p < 0.0001) were the only proteins with significantly reduced FliW binding (shown in red). Mean and SD of three biological replicates (separate colonies) are represented. One-way ANOVA with Dunnett’s multiple comparisons test was used for statistical analysis (using GraphPad Prism software). Three independent experiments were performed. Daggers indicate amino acids that are involved in RNA binding.

To further refine the FliW binding site, we used a combination of SDM and a bacterial two-hybrid system. In this system, potentially interacting proteins are constructed as fusion proteins with two domains (T18 and T25) of the *B. pertussis* adenylate cyclase toxin (Karimova et al., 1998). Positive interaction reconstitutes cAMP synthesis and results in β-galactosidase activity in the *E. coli* reporter strain Sp850 (Karimova et al., 1998). We first cloned the *C. jejuni csrA* gene into one of the two hybrid vectors (pT18) and *fliW* in the other (pT25) (Figure 2A). Compared to a control strain containing pT18 and pT25 only, co-expression of pT18-*csrA* and pT25-*fliW* generated significant β-galactosidase activity, indicating positive interaction in this system (Figure 2B). We next performed SDM on *C. jejuni csrA* cloned into pT18, changing each amino acid to alanine (except for two native alanine residues, which were changed to valines). Each site-directed mutant was introduced into *E. coli* Sp850 containing pT25-*fliW*. β-galactosidase activities showed that 73 of 75 site-directed mutants had interactions of CsrA and FliW that were not significantly different than WT (Figure 2B). However, CsrA-V51A and CsrA-N55A showed a significant reduction in β-galactosidase activity, demonstrating that these two amino acids are required for binding of FliW to CsrA (Figure 2B). These amino acids lie just C-terminally to amino acids involved in RNA binding by *C. jejuni* CsrA (Figure 2B; El Abbar et al., 2019).

### CsrA V51A and N55A Have Decreased Affinity to FliW

**FIGURE 3:**
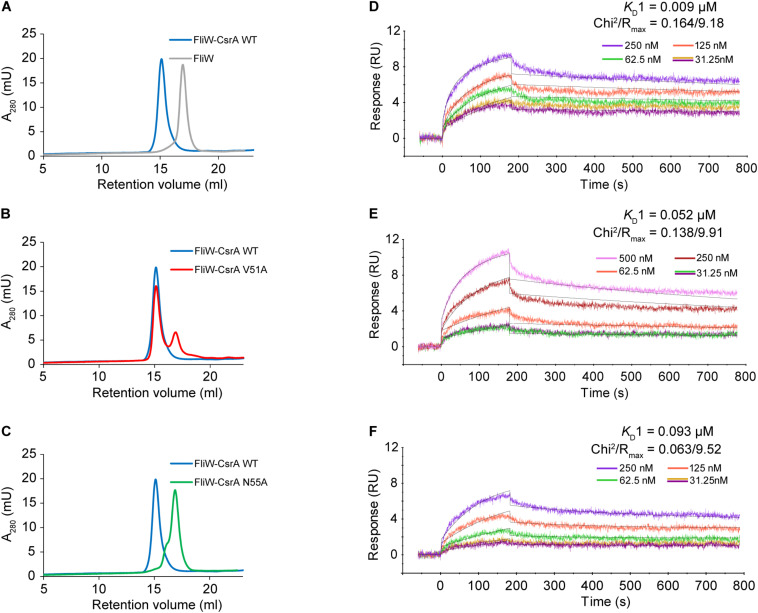
Analysis of the interaction between WT and mutant CsrA with FliW by analytical SEC and SPR. **(A–C)** The association of FliW with WT and site-directed mutants of CsrA was analyzed using analytical SEC. Purified FliW-His_6_ and CsrA-WT-His_6_ formed a stable CsrA-FliW complex [**(A)** blue tracing]. FliW-His_6_ and CsrA-V51A-His_6_ resulted in a mixture of a complex and unbound proteins [**(B)** red tracing], demonstrating reduced interaction. Presence of the complex was not observed for the FliW-His_6_ and CsrA-N55A-His_6_ mixture [**(C)** green tracing], indicating a lack of interaction between the two proteins. **(D–F)** Affinity of interaction between FliW and CsrA (WT, V51A, N55A) was determined based on SPR kinetic assay. FliW-His_6_ was immobilized to the sensor chip. The association was made by injecting different concentrations (0–500 nM) of CsrA-His_6_ (WT, V51, and N55A) sample at a constant flow rate of 30 μL/min for 180 s, followed by 600 s of dissociation. The dissociation constant was determined for the first binding event (*K*_*D*_1).

To confirm that CsrA V51 and N55 were involved in binding FliW, we evaluated the protein-protein interactions of FliW with WT and mutant CsrA, using two *in vitro* methods. In size-exclusion chromatography experiments, we passed purified FliW-His_6_ and CsrA-His_6_ (WT, V51A, or N55A) either alone, or mixed in equimolar ratios over a SEC column. FliW-His_6_ eluted at a retention volume of 16.9 ml (Figure 3A). Mixture of FliW-His_6_ and CsrA-WT-His_6_ resulted in a stable heterotetramer of CsrA_2_-FliW_2_ (Li et al., 2018), with a retention volume of 15.1 ml (Figure 3A). FliW-His_6_ and CsrA-V51A-His_6_ gave mixture of protein complex and unbound proteins, indicating weaker interaction (Figure 3B). No complex was formed between FliW-His_6_ and CsrA-N55A-His_6_ in the equimolar mixture of proteins, indicating lack of protein-protein interaction (Figure 3C). These results are consistent with the findings from two-hybrid experiments (Figure 2B). To further determine the affinities of CsrA WT, V51A, and N55A to FliW, we performed binding assays using surface plasmon resonance. Purified FliW-His_6_ was immobilized onto the chip surface and different CsrA-His_6_ (WT, V51A, or N55A) concentrations were run over the chip surface to obtain binding parameters (Figures 3D–F). Data followed a bivalent analyte binding model, confirming that one molecule of CsrA dimer is able to bind two molecules of FliW. The *K*_*D*_ for the first ligand binding site of CsrA-WT was calculated to be 9 × 10^–9^ M, showing strong binding (Figure 3D). As expected from the SEC experiment, the affinity of CsrA-V51A to FliW was reduced 6-fold (*K*_*D*_ = 5.2 × 10^–8^ M), while binding of CsrA-N55A was decreased 10-fold (*K*_*D*_ = 9.3 × 10^–8^ M) (Figures 3E,F). Taken together, our results from the two-hybrid assay and both *in vitro* binding assays indicate that amino acids V51 and N55 are crucial for full FliW binding by CsrA.

### CsrA V51 and N55 Are Conserved Among Diverse Bacteria Species

**FIGURE 4:**
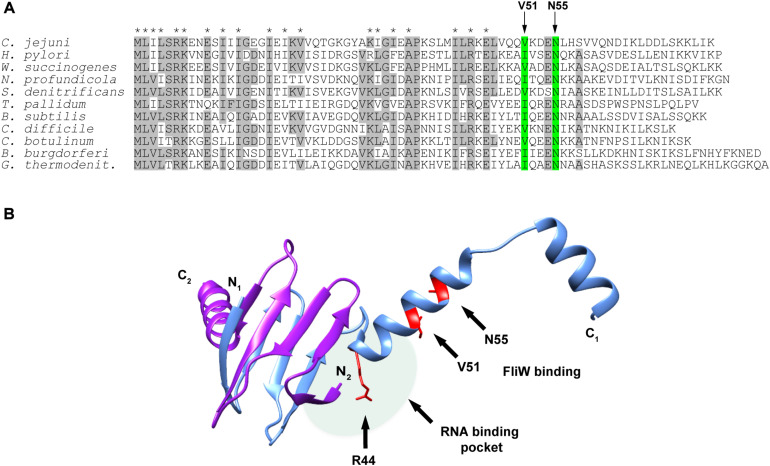
Alignment of CsrA protein sequences from diverse FliW-containing bacterial species and molecular model showing the location of the FliW binding site. **(A)** CsrA proteins from 11 bacterial species that have CsrA with extended C-terminal regions and that also possess FliW were aligned using ClustalOmega. Amino acids that were identical in at least 6 of 11 of these proteins are shaded gray. Asterisks above the alignment indicate amino acids that were shown to be involved in RNA binding by *C. jejuni* CsrA (El Abbar et al., 2019). Arrows and green shading show the locations of V51 and N55 that are involved in binding of FliW to *C. jejuni* CsrA. **(B)** The crystal structure of the CsrA ortholog from *G. thermodenitrificans* (Altegoer et al., 2016) was used to model the location of the FliW binding site. CsrA is an antiparallel homodimer in which two RNA-binding pockets are formed between the β_1_ and β_5_ sheets of opposing subunits (Figure 1A; Mercante et al., 2006). One RNA binding pocket is shaded (pale green oval); the second pocket on the opposite side of the dimer is not shown for clarity of the figure. Amino acids V51 and N55 (shown in red) that are involved in FliW binding are predicted to be located immediately adjacent to the RNA-binding pockets containing R44, which is crucial for RNA binding. The figure was generated using UCSF Chimera (Pettersen et al., 2004).

We next examined CsrA proteins for conservation of these FliW-binding amino acids among diverse bacteria expressing CsrA proteins with extended C-terminal regions and containing FliW. Comparison of these proteins showed that N55 was identical in CsrA proteins from all species examined (Figure 4A). V51 was identical in five of the species examined, while in the other five species this position was occupied by the conservative substitution isoleucine.

### FliW-Binding Amino Acids of CsrA Are Not Involved in CsrA-mRNA Interaction

A crystal structure of *C. jejuni* CsrA is not currently available. To model CsrA-FliW interactions, we used the published structure of CsrA from *G. thermodenitrificans* (Altegoer et al., 2016; Figure 4B). The CsrA proteins from *G. thermodenitrificans* and *C. jejuni* are 38% identical/60% similar, and both have extended C-terminal regions compared to *E. coli* CsrA (Fields and Thompson, 2012). Validity of this model was further emphasized by the fact that CsrA amino acid residues V51 and N55 correspond to isoleucine and asparagine in the CsrA ortholog from *G. thermodenitrificans*, respectively. According to this structure, a symmetrical homodimer of CsrA binds two molecules of FliW on the opposite sides of the molecule. The amino acids with roles in FliW binding lie within the first α-helix C-terminally adjacent to the RNA-binding site. These are distinct from residues that were shown previously to play a role in mRNA binding by *C. jejuni* CsrA, which mainly occupy positions within the β-strands in the core of the CsrA homodimer (Figures 1A, 4B; El Abbar et al., 2019). They are however predicted to be located adjacent to the RNA-binding groove at the edge of the CsrA dimer β-sheet, which in this model is adjacent to the aforementioned α-helix (Figure 4B). As amino acids involved in interaction with FliW seem not to be necessary for mRNA binding, we propose that FliW inhibition of CsrA regulatory activity does not rely on direct competition for the CsrA RNA-binding site.

## Discussion

We previously showed that CsrA is a pleiotropic post-transcriptional regulator in *C. jejuni*, modulating the expression of numerous proteins including FlaA flagellin (Fields and Thompson, 2008; Fields et al., 2016; Li et al., 2018). In multiple bacteria including *C. jejuni*, CsrA regulatory activity is controlled by protein-protein interactions with FliW (Mukherjee et al., 2011, 2013; Dugar et al., 2016; Fields et al., 2016; Radomska et al., 2016; Li et al., 2018). In *B. subtilis*, homeostatic control of flagellin (Hag) expression is achieved by maintaining a 1:1:1 ratio of Hag:FliW:CsrA that ensures proper expression of Hag subunits during flagellar synthesis (Oshiro et al., 2019). In *C. jejuni*, mutation of *fliW* results in decreased synthesis of flagellin, while a double *fliW* and *csrA* mutation leads to increased flagellin levels (Dugar et al., 2016; Radomska et al., 2016; Li et al., 2018). A *C. jejuni fliW* mutant has markedly shortened flagella, but its length is restored in the double *fliWcsrA* mutant (Dugar et al., 2016; Li et al., 2018). This clearly indicates that the defect of flagellar synthesis is dependent on the regulatory interplay between these two proteins, rather than the lack of FliW chaperone activity. Furthermore, FliW was the first protein described to interact with CsrA (Mukherjee et al., 2011), representing a novel mechanism of CsrA regulation by protein partners rather than sRNAs. The mechanism by which *C. jejuni* FliW antagonizes CsrA inhibitory activity remains poorly understood, therefore the aim of this study was to define the protein-protein interactions between *C. jejuni* CsrA and FliW. Here we show that in *C. jejuni* CsrA binds FliW at a site adjacent to its predicted RNA-binding sites, thus inhibiting CsrA regulatory activity on target mRNAs.

Alignment of CsrA proteins from diverse bacteria revealed that CsrA in *C. jejuni*, *H. pylori*, and other bacteria in which CsrA-regulating sRNAs are not present have an extended C-terminal region (Fields and Thompson, 2012). We reasoned that this was a likely site for interaction with FliW as it was previously indicated in *G. thermodenitrificans* (Altegoer et al., 2016). C-terminal deletion analysis showed that although somewhat unexpectedly the deletion of 15 amino acids had no appreciable effect on FliW binding, the interaction occurs in this region but not strictly within the extension itself (Figure 1). The Δ20 deletion (deletion of CsrA amino acids 56–75) showed greatly reduced FliW binding, despite significant expression of CsrA Δ20 in the *E. coli* lysates used for the pulldowns (data not shown). Nevertheless, the deletion studies defined amino acids 61–75 of *C. jejuni* CsrA as non-essential for FliW binding, and suggested that amino acids 56–61 were at least adjacent to the binding site. To comprehensively determine which amino acids were crucial for FliW binding, we used a bacterial two-hybrid system and saturating alanine scanning mutagenesis to assess CsrA-FliW interaction. In these studies, we identified two residues, V51 and N55, as crucial for full FliW binding (Figure 2B). These two amino acids actually lie just N-terminal to the CsrA C-terminal amino acids that were deleted in the binding experiments presented in Figure 1. This suggests that while N55 is critical for binding, the location of N55 at the extreme C-terminus of the truncated protein does not allow full binding. This is likely because the local structural context of CsrA N55 must be maintained for full binding of FliW to occur (see also below).

To substantiate the roles of CsrA V51 and N55 in binding to FliW, these findings were further confirmed *in vitro* using gel filtration of equimolar mixtures of CsrA (WT, V51A, or N55A) with FliW (Figures 3A–C) and by assessing the binding affinities with surface plasmon resonance (Figures 3D–F). We show that the affinity of WT CsrA-FliW interaction is high such that in an equimolar mixture of the two proteins, the entire fraction of FliW is complexed with CsrA (Figures 3A,D). While the V51A mutation showed a 6-fold reduction of affinity, the N55A mutation led to a strong reduction of the binding strength (10-fold) which resulted in undetectable levels of the complex *in vitro* (Figures 3C,F).

Those *in vitro* data align with the degree of FliW binding inhibition observed in two-hybrid experiments. We found that V51 and especially N55 are highly conserved among species in which CsrA contains the C-terminal extension, with N55 being conserved in all analyzed species and V51 being either identical or conservatively substituted with isoleucine (Figure 4A; Fields and Thompson, 2012). Furthermore, the importance of residue N55 in FliW binding was originally shown in the phylogenetically distant *B. subtilis*, where mutation of this highly conserved asparagine to aspartic acid prevented FliW binding by CsrA (Mukherjee et al., 2016).

Although the structure of *C. jejuni* CsrA is not yet available, structural details of a CsrA-FliW complex have been studied in *G. thermodenitrificans* (Altegoer et al., 2016). In this structure, I52 and N56 (corresponding to *C. jejuni* V51 and N55, respectively) lie in the middle of the first α-helix and are part of the CsrA-FliW interface. Such a localization of those residues in *C. jejuni* CsrA could further explain why it was observed that the deletion of 20 C-terminal amino acids abrogates FliW binding despite the fact that amino acids crucial for binding do not lie within the deleted segment. It is possible that this deletion disrupts the structure of the α-helix and, although the crucial N55 residue is left as the C-terminal residue in the Δ20 mutant, the deletion renders the remaining structure unable to bind FliW to an appreciable extent.

We previously identified *C. jejuni* CsrA amino acids responsible for binding to one of its major targets, *flaA* mRNA (El Abbar et al., 2019). Interestingly, none of those amino acids were shown in the current study to contribute in any degree to CsrA association with FliW. In that study, most of the amino acids with significant roles in RNA binding clustered within the predicted β-strands of CsrA (including β_1_ and β_5_ which form the RNA-binding pocket) rather than the α-helical, C-terminal portion of the molecule, where FliW binding occurs (Figure 4A; El Abbar et al., 2019). In the known structures of CsrA orthologs, strands β_1_ and β_5_ are located on the same edge of the inter-subunit β-sheet (Gutierrez et al., 2005; Rife et al., 2005; Heeb et al., 2006; Schubert et al., 2007) and positioned adjacent to the α-helix. Binding of target mRNA at this position was observed in the known NMR structure of the CsrA ortholog (RsmE) from *P. fluorescens*, although RsmE is devoid of the C-terminal extension (Schubert et al., 2007).

All of these observations suggest that FliW binds CsrA adjacent to but at a different site than its RNA-binding site. However, in the absence of further structural details, the mechanism by which FliW antagonizes CsrA in *C. jejuni* remains incompletely understood. The binding of FliW to CsrA at amino acids V51 and N55 could diminish CsrA activity in a few ways. First, although FliW uses different CsrA amino acids to create the complex, binding within the α-helix immediately adjacent to the RNA-binding pockets of CsrA could sterically occlude the entry of target mRNAs. The protein chaperone CesT was shown to antagonize *E. coli* CsrA in this manner by binding to the site which significantly overlaps with the mRNA binding site (Katsowich et al., 2017; Ye et al., 2018). Second, the negatively charged surface of FliW near the RNA-binding interface could electrostatically repel the phosphate backbone of the RNA, as suggested for *G. thermodenitrificans* CsrA (Altegoer et al., 2016), especially considering the proximity of V51 and N55 to R44, which is critical for RNA binding (Mercante et al., 2006; El Abbar et al., 2019; Figure 4B). Finally, an allosteric mechanism with FliW binding to the separate allosteric surface has also been suggested in *B. subtillis* (Mukherjee et al., 2016), where FliW binding was similarly shown not to require the mRNA binding residues of CsrA (Altegoer et al., 2016).

In summary, *C. jejuni* controls the expression of numerous metabolic and virulence-related proteins by means of the CsrA-FliW regulatory system. The role of FliW in regulating CsrA activity and the expression of FlaA links *C. jejuni* metabolism with its critical virulence factor of cellular motility and invasion. Our results define the nature of the *C. jejuni* CsrA-FliW interaction and open the possibility of targets for therapeutic intervention.

## Data Availability Statement

All datasets generated for this study are included in the article/Supplementary Material.

## Author Contributions

MB, FE, CC, JL, JF, LT, PT, CD, KB, ZW, PA, and ST contributed to the conception and design of the study, and performed experiments described in the manuscript. MB wrote the first draft of the manuscript. MB, FE, and ST wrote sections of the manuscript. All authors contributed to manuscript revision, read and approved the submitted version.

## Conflict of Interest

The authors declare that the research was conducted in the absence of any commercial or financial relationships that could be construed as a potential conflict of interest.
