# Supplementary material for: Binding of Campylobacter jejuni FliW Adjacent to the CsrA RNA-Binding Pockets Modulates CsrA Regulatory Activity
Source: Front Microbiol. 2021 Jan 11;11:531596. doi: 10.3389/fmicb.2020.531596 (PMC7829508; doi:10.3389/fmicb.2020.531596)
Supplement: Supplementary file 1 [file Table_1.pdf]

Table S1. PCR primers used

Primer	Sequence (5' -> 3')	Mutation or description
**Forward**		
csrA-del.F	CCCCTCGAGCACCACCACCA	F for C-terminal deletions
csrA-KpnI.F	GGAGGTACCGATGTTAATATTATCAAGAAAAGAAAATGA	F for cloning mutants 40-60
csrA.M1A.F	GGAGGTACCGGCGTTAATATTATCAAGAAAAGAAAATGA	M1A
csrA.L2A.F	GGAGGTACCGATGGCAATATTATCAAGAAAAGAAAATGA	L2A
csrA.I3A.F	GGAGGTACCGATGTTAGCATTATCAAGAAAAGAAAATGA	I3A
csrA.L4A.F	GGAGGTACCGATGTTAATAGCATCAAGAAAAGAAAATGA	L4A
csrA.S5A.F	GGAGGTACCGATGTTAATATTAGCAAGAAAAGAAAATGA	S5A
csrA.R6A.F	GGAGGTACCGATGTTAATATTATCAGCAAAAGAAAATGA	R6A
csrA.K7A.F	GGAGGTACCGATGTTAATATTATCAAGAGCAGAAAATGAAAGTAT	K7A
csrA.E8A.F	GGAGGTACCGATGTTAATATTATCAAGAAAAGCAAATGAAAGTAT	E8A
csrA.N9A.F	GGAGGTACCGATGTTAATATTATCAAGAAAAGAAGCTGAAAGTAT	N9A
csrA.E10A.F	GGAGGTACCGATGTTAATATTATCAAGAAAAGAAAATGCAAGTAT	E10A
csrA.S11A.F	AGCTATAATTATCGGAGAAGGTATAG	S11A
csrA.I12A.F	AAGTGCAATTATCGGAGAAGGTATAG	I12A
csrA.I13A.F	AAGTATAGCTATCGGAGAAGGTATAG	I13A
csrA.I14A.F	AAGTATAATTGCCGGAGAAGGTATAG	I14A
csrA.G15A.F	CGCAGAAGGTATAGAAATCAAAG	G15A
csrA.E16A.F	CGGAGCAGGTATAGAAATCAAAG	E16A
csrA.G17A.F	CGGAGAAGCTATAGAAATCAAAG	G17A
csrA.I18A.F	CGGAGAAGGTGCAGAAATCAAAG	I18A
csrA.E19A.F	GCAATCAAAGTCGTTCAAACAGG	E19A
csrA.I20A.F	GAAGCCAAAGTCGTTCAAACAGG	I20A
csrA.K21A.F	GAAATCGCAGTCGTTCAAACAGG	K21A
csrA.V22A.F	GAAATCAAAGCCGTTCAAACAGG	V22A
csrA.V23A.F	GCTCAAACAGGGAAAGGATATGC	V23A
csrA.Q24A.F	GTTGCAACAGGGAAAGGATATGC	Q24A
csrA.T25A.F	GTTCAAGCAGGGAAAGGATATGC	T25A
csrA.G26A.F	GTTCAAACAGCGAAAGGATATGC	G26A
csrA.K27A.F	GCAGGATACGCAAAAATAGGAATAGAAGC	K27A
csrA.G28A.F	ACAGGGAAAGCTTATGCCAAAATAG	G28A
csrA.Y29A.F	AGGGAAAGGAGCTGCCAAAATAGG	Y29A
csrA.A30V.F	AAAGGATATGTCAAAATAGGCATAG	A30V
csrA.K31A.F	CGCAATAGGAATAGAAGCTCCAAAATC	K31A
csrA.I32A.F	CAAAGCAGGCATAGAAGCTCCAAAATC	I32A
csrA.G33A.F	CAAAATAGCCATAGAAGCTCCAAAATC	G33A
csrA.I34A.F	CAAAATAGGCGCAGAAGCTCCAAAATC	I34A
csrA.E35A.F	AGCAGCTCCAAAATCCCTTATGATAC	E35A
csrA.A36V.F	AGAAGTTCCAAAATCCCTTATGATAC	A36V
csrA.P37A.F	AGAAGCTGCAAAATCCCTTATGATAC	P37A
csrA.K38A.F	AGAAGCTCCAGCATCCCTTATGATACTTAGAAAA	K38A
csrA.S39A.F	AGCTCCAAAGGCCCTTATGATAC	S39A
csrA.Q61A.F	TGCGAATGATATTAAACTTGATGACTTAAG	Q61A
csrA.N62A.F	TCAGGCTGATATTAAACTTGATGACTTAAG	N62A
csrA.D63A.F	TCAGAATGCTATTAAACTTGATGACTTAAG	D63A
csrA.I64A.F	TCAGAATGATGCTAAACTTGATGACTTAAG	I64A
csrA.K65A.F	ATTGCACTTGATGACTTAAGCAAAAAAC	K65A
csrA.L66A.F	ATTAAAGCTGATGACTTAAGCAAAAAAC	L66A
csrA.D67A.F	ATTAAACTTGCTGACTTAAGTAAAAAAC	D67A
fliW-PstI.F	GGGCTGCAGGGATGACCCTAGCTGTTAAATGC	FliW cloning
**Reverse**		
csrA-5.R	AAACTCGAGGCTTAAGTCATCAAGTTT	CsrAΔ5
csrA-10.R	AAACTCGAGTTTAATATCATTCTGAAC	CsrAΔ10

csrA-15.R	AAACTCGAGAACAACAGAATGCAAATT	CsrAΔ15
csrA-20.R	AAACTCGAGATTTTCATCCTTTACTTGTTG	CsrAΔ20
csrA-25.R	AGTTCTTTTCTAAGTATCATAAG	CsrAΔ25
csrA-HindIII.R	CCCAAGCTTCCTTTGATTAGTTTTTTGCTTAAGTC	R for cloning mutants 40-60
csrA.11-14.R	TCATTTTCTTTTCTTGATAATATTAAC	R for mutants 11-14
csrA.15-18.R	ATAATTATACTTTCATTTTCTTTTCTTG	R for mutants 15-18
csrA.19-22.R	TATACCTTCTCCGATAATTATAC	R for mutants 19-22
csrA.23-26.R	GACTTTGATTTCTATACCTTC	R for mutants 23-26
csrA.27&30.R	CCCTGTTTGAACGACTTTG	R for mutants 27&30
csrA.28.R	TTGAACGACTTTGATTTCTATAC	R for mutant 28
csrA.29.R	GTTTGAACGACTTTGATTTC	R for mutant 29
csrA.31-34.R	GCATATCCTTTCCCTGTTTG	R for mutants 31-34
csrA.35-38.R	ATTCCTATTTTGGCATATC	R for mutants 35-38
csrA.39.R	TCTATGCCTATTTTTGCGTATCCTTTCCC	R for mutant 39
csrA.61-64.R	ACAACAGAATGCAAGTTTTCATCCTT	R for mutants 61-64
csrA.65-67.R	ATCGTTTTGAACAACAGAATG	R for mutants 65-67
csrA.D68A.R	CCCAAGCTTCCTTTGATTAGTTTTTTGCTTAAG**GC**ATCAAG	D68A
csrA.L69A.R	CCCAAGCTTCCTTTGATTAGTTTTTTGCTT**GC**GTCATCAAG	L69A
csrA.S70A.R	CCCAAGCTTCCTTTGATTAGTTTTTTG**GC**TAAGTCATCAAG	S70A
csrA.K71A.R	CCCAAGCTTCCTTTGATTAGTTTT**GC**GCTTAAGTC	K71A
csrA.K72A.R	CCCAAGCTTCCTTTGATTAGT**GC**TTTGCTTAAGTC	K72A
csrA.L73A.R	CCCAAGCTTCCTTTGATT**GC**TTTTTTGCTTAAGTC	L73A
csrA.I74A.R	CCCAAGCTTCCTTTG**GC**TAGTTTTTTGCTTAAGTC	I74A
csrA.K75A.R	CCCAAGCTTCCT**GC**GATTAGTTTTTTGCTTAAGTC	K75A
fliW-BamHI.R	GGGGGATCCAATTTTTTAATATAATTAGCAATTTGATCAGCTTGA	FliW cloning

Nucleotides encoding amino acid substitutions are indicated by bold shading with single underline. Synonymous nucleotide substitutions made to decrease secondary structure in PCR primers are indicated by double underlines. Restriction enzyme sites for cloning purposes are indicated by wavy underlines.
